# Isotopic Niche Analysis of Long-Finned Pilot Whales (*Globicephala melas edwardii*) in Aotearoa New Zealand Waters

**DOI:** 10.3390/biology11101414

**Published:** 2022-09-28

**Authors:** Bethany Hinton, Karen A. Stockin, Sarah J. Bury, Katharina J. Peters, Emma L. Betty

**Affiliations:** 1 Cetacean Ecology Research Group, School of Natural Sciences, Massey University, Auckland 0745, New Zealand; 2 Environmental Isotopes and Molecular Biology Group, National Institute of Water and Atmospheric Research, Wellington 6021, New Zealand; 3 School of Earth and Environment, University of Canterbury, Christchurch 8041, New Zealand; 4 Evolutionary Genetics Group, Department of Anthropology, University of Zurich, 8057 Zurich, Switzerland; 5 Global Ecology, College of Science and Engineering, Flinders University, Adelaide, SA 5001, Australia

**Keywords:** trophic ecology, foraging ecology, isotope, *δ*^13^C, *δ*^15^N, *δ*^34^S

## Abstract

**Simple Summary:**

Isotopic niche analyses can elucidate a species’ foraging ecology. Using isotopic values of *δ*^13^C, *δ*^15^N and *δ*^34^S, the isotopic niche of long-finned pilot whales (*Globicephala melas edwardii*) in Aotearoa New Zealand was investigated for animals that stranded in six different events across two locations between 2009 and 2017. Generalised additive models revealed that stranding event was a stronger predictor for *δ*^13^C and *δ*^15^N values than body length, sex, or reproductive status, indicating that spatiotemporal differences explained isotopic variation of *G. m. edwardii* in New Zealand waters better than ontogenetic factors.

**Abstract:**

The quantification of a species’ trophic niche is important to understand the species ecology and its interactions with the ecosystem it resides in. Despite the high frequency of long-finned pilot whale (*Globicephala melas edwardii*) strandings on the Aotearoa New Zealand coast, their trophic niche remains poorly understood. To assess the isotopic niche of *G. m. edwardii* within New Zealand, ontogenetic (sex, total body length, age, maturity status, reproductive group) and spatiotemporal (stranding location, stranding event, and stranding year) variation were investigated. Stable isotopes of carbon (*δ*^13^C) and nitrogen (*δ*^15^N) were examined from skin samples of 125 *G. m. edwardii* (67 females and 58 males) collected at mass-stranding events at Onetahua Farewell Spit in 2009 (*n* = 20), 2011 (*n* = 20), 2014 (*n =* 27) and 2017 (*n =* 20) and at Rakiura Stewart Island in 2010 (*n =* 19) and 2011 (*n =* 19). Variations in *δ*^34^S values were examined for a subset of 36 individuals. General additive models revealed that stranding event was the strongest predictor for *δ*^13^C and *δ*^15^N values, whilst sex was the strongest predictor of *δ*^34^S isotopic values. Although similar within years, *δ*^13^C values were lower in 2014 and 2017 compared to all other years. Furthermore, *δ*^15^N values were higher within Farewell Spit 2017 compared to any other stranding event. This suggests that the individuals stranded in Farewell Spit in 2017 may have been feeding at a higher trophic level, or that the nitrogen baseline may have been higher in 2017 than in other years. Spatiotemporal differences explained isotopic variation of *G. m. edwardii* in New Zealand waters better than ontogenetic factors.

## 1. Introduction

Stable isotope analysis has steadily grown as an ecological tool over recent years [1], with the method now commonly applied to trophic analysis and foraging ecology [2,3]. For example, stable isotopes have been used to determine dietary niche and relative prey contribution to diet for a wide range of marine and freshwater species [4,5,6,7,8], including cetaceans (whales, dolphins, and porpoises; [9,10]).

Multiple isotopes have been used in foraging research including carbon [11,12], nitrogen [13], oxygen [14], sulphur [12], and strontium [15]. Isotopic values of carbon are typically used to infer information relating to foraging habitat [11,16,17], whereas nitrogen isotopes have been linked to protein quantity, quality, and trophic feeding level [18,19]. Sulphur isotopes (*δ*^34^S) combined with carbon (*δ*^13^C) and nitrogen (*δ*^15^N) isotopes, are now increasingly being used to provide clarity around prey source pathways, e.g., estuarine or marine [20]. The combination of these isotopes can elucidate approximate feeding habitats, trophic level source and food web pathways, and provide information on the isotopic niche of an animal. Triple isotope studies have been successfully used in studies of marine ecosystems [20,21], including cetacea [22,23,24], especially to describe isotopic niche. Whilst isotopic niche should be considered as a distinct entity from trophic niche [25,26], the two are likely correlated [27]. Hence, isotopic niche can be used to help describe trophic niche, given correct consideration of the ecological context [28].

Trophic niche partitioning between species is a common strategy to reduce resource competition [29,30]. Isotopic niche differences have been observed between different cetacean species inhabiting the same geographical area [10,31,32,33,34,35]. This reduction in foraging competition could also be driving isotopic niche differences within socially distinct populations of the same species [36,37] and even between individuals within the same population [38]. Isotopic variation within a population has been linked to ontogenetic factors such as age [24,39], sex [40], total body length (herein referred to as “body length”; [41,42]), life stage [43], or sexual maturity status [44]. Although some species have shown isotopic homogeneity within a population [45], diet may still change between spatially or socially distinct populations of the same species as is observed in killer whale *Orcinus orca* [40], bottlenose dolphins *Tursiops truncatus* [46] and long-finned pilot whales *Globicephala melas* [47].

Whilst both spatial and seasonal differences in *G. m. melas* isotopic values have been noted [37,47,48], dietary differences have also been reported to be related to body size [48,49]. In New Zealand, the southern hemisphere long-finned pilot whale subspecies *G. m. edwardii* is the most frequently stranded cetacean by number and several locations have been identified as local stranding hotspots [50]. Stomach content analyses of 37 *G. m. edwardii* from three stranding events in New Zealand described six cephalopod species present in their stomachs [51,52,53]. Whilst stomach content analysis provides important short-term dietary insights [54,55], it does not give information on diet that has already been assimilated over a longer timescale, which can be provided through isotopic investigation [56]. Furthermore, insights to intraspecific dietary or trophic variation and local isotopic niche of this sub-species are also lacking. In this study, we aimed to address some of these knowledge gaps by exploring ontogenetic and spatiotemporal variation in isotopic niche for *G. m. edwardii* from two stranding hotspots in New Zealand. Specifically, we investigated (1) the isotopic niche of *G. m. edwardii* in New Zealand using carbon, nitrogen, and sulphur isotopes, (2) ontogenetic variation in isotope values by sex, body length, age, maturity status and reproductive group and (3) spatiotemporal overlap in isotopic niche. 

## 2. Materials and Methods

To assess isotopic profiles of *G. m. edwardii* in New Zealand waters, archived skin samples *(n =* 125) were analysed from individuals collected from stranding events between 2009 and 2017 (summarised in Supplementary Material Table S1). 

### 2.1. Sampling

**Figure 1:**
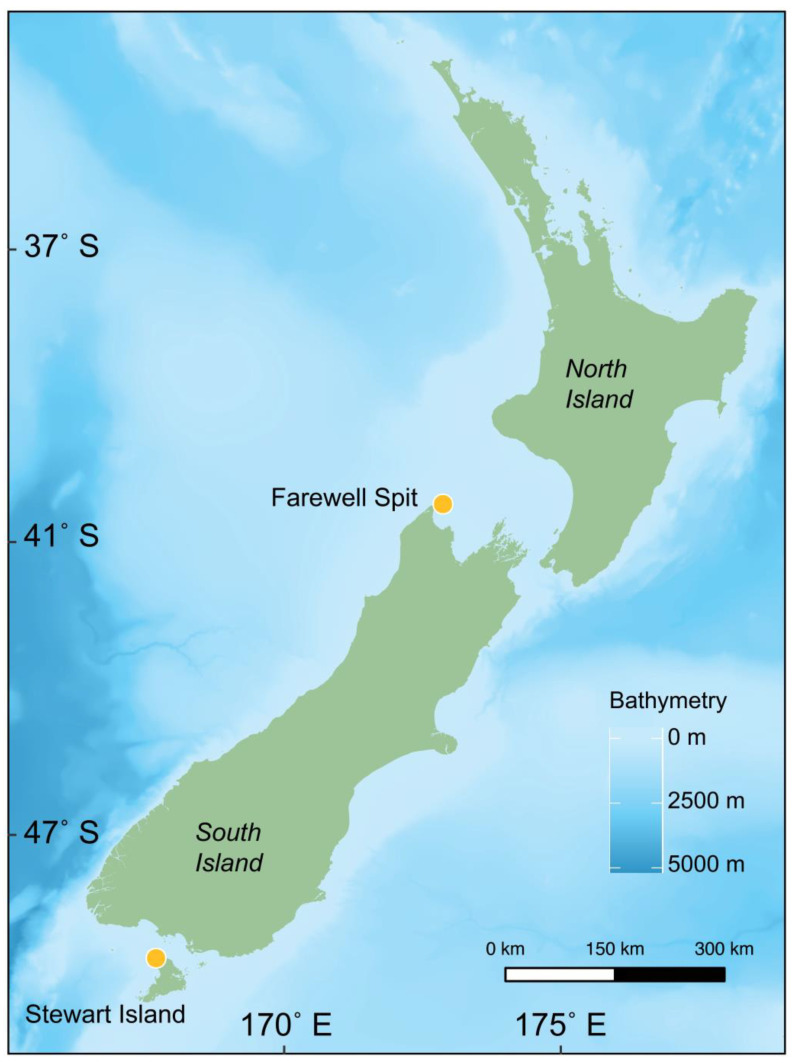
Location of sampling sites of long-finned pilot whale (*Globicephala melas edwardii*) carcasses from mass-stranding events at Farewell Spit and Stewart Island, Aotearoa New Zealand. Bathymetry is depicted with darker shades of blue representing deeper waters (reprinted with permission from National Institute of Water and Atmospheric Research (NIWA) under a Creative Commons BY license, with permission from NIWA original copyright [57].

Skin was sampled from six stranding events across two *G. m. edwardii* stranding hotspot locations in New Zealand; Onetahua Farewell Spit (FWS; −40.481° S, 172.870° E) and Rakiura Stewart Island (SI; −46.686° S, 167.685° E; [50]; see Figure 1). Of these, 87 carcasses were sampled at FWS during four mass-stranding events (2009, 2011, 2014, 2017) and 38 carcasses at SI during two mass-stranding events (2010, 2011). All of the mass-strandings sampled occurred during the austral summer between the months of November and February.

Skin sampling, along with measurements of body length and an anatomical assessment of sex, was undertaken in situ at stranding events using standard postmortem procedures [58]. All skin samples were stored at 4 °C in 70% ethanol prior to analysis. Teeth and reproductive organs were sampled where possible, as outlined in [59], with age data available for 86% (108 of 125) of individuals and reproductive data available for 82% (102 of 125) individuals. Teeth were used to assess age via dentinal growth layer groups [59]. Reproductive organs were used to assess sexual maturity status (herein referred to as maturity status) and reproductive group for mature females, where possible [60,61]. Six reproductive groups were defined: immature males, mature males, immature females, pregnant females, lactating females, and resting females. Male maturity was defined by presence/absence of sperm in testes [61]. Females were defined as “pregnant” by the presence/absence of a foetus, as “lactating” by presence/absence of milk in the mammary glands, and as “resting” by the presence of ovarian corpora indicating previous ovulation, but with no foetus or milk present [60]. However, if reproductive group and/or maturity status were not available, body length was used as an indicator of maturity status using estimations from the same *G. m. edwardii* population [60,61]. Where sample availability allowed, samples were compared in equal groups of mature males (*n =* 5), mature females (*n =* 5), immature males (*n =* 5) and immature females (*n =* 5) within each stranding event. In one stranding event (FWS2014), more mature females of known reproductive group were available, and these were therefore included in analyses to increase comparative statistical power of mature female reproductive groups (Table 1). 

Carbon and nitrogen stable isotopes from skin samples (*n* = 125) were analysed to compare ontogenetic and spatiotemporal variation. Additionally, a subset of 36 (13 male and 23 female) samples from sexually mature individuals with the highest, lowest, and median carbon and nitrogen isotope values recorded per stranding event were analysed for sulphur isotope values. Immature individuals excluded from analyses of sulphur isotopes to avoid confounding the data with individuals that were not fully weaned.

### 2.2. Sample Preparation

In preparation for stable isotope analysis, skin samples were placed under the fume hood for at least 48 h [62] to evaporate off the storage ethanol. Samples with excess ethanol remaining were further placed under a stream of nitrogen gas until all ethanol had been removed from the sample. Samples were cut longitudinally to capture all skin layers, as recommended for isotopic studies of cetaceans aiming to consider trophic interactions and diet composition [63]. Skin was then homogenized by finely slicing in a glass Petri dish using a clean scalpel blade. Approximately 40 mg of each sample was weighed into Eppendorf tubes and freeze-dried overnight for a minimum of 18 h or dried in an oven at 60 °C for at least 48 h. 

### 2.3. Carbon and Nitrogen Isotope Analysis

Carbon and nitrogen isotope analysis was carried out at the Environmental and Ecological Stable Isotope Analytical Facility at the National Institute for Water and Atmospheric Research (NIWA), Wellington. Around 1.0 mg of each homogenised skin sample was weighed into tin capsules using a six decimal place (g) microbalance. Tin capsules were formed into balls containing the sample and were analysed by a FLASH 2000 elemental analyser with MAS 200 R autosampler linked to a DELTA V Plus continuous flow isotope ratio mass spectrometer (Thermo Fisher Scientific, Bremen, Germany). Stable isotope values were calculated using ISODAT (Thermo Fisher Scientific) software; *δ*^13^C values were calibrated against Carrara Marble NSB-19 (National Institute of Standards and Technology (NIST), Gaithersburg, MD, USA) and *δ*^15^N relative to Pee Dee Beleminte (PDB) standard followed by correction for O^17^. International laboratory reference materials from NIST were run at the start and end of eve ry batch of analyses for data normalisation [64]. A working laboratory standard of DL-Leucine (DL-2-Amino-4-methylpentanoic acid, C6H13NO2, Lot 127H1084, Sigma, Melbourne, Australia) and squid were run every 10 samples to correct for machine drift, for quality control and to report on precision. The international standards USGS65 Glycine was also run every ten samples to check accuracy and precision. Data accuracy was measured to better than 0.15‰ for *δ*^13^C and *δ*^15^N values, whilst precision was measured to better than 0.24‰ for *δ*^13^C and 0.22‰ for *δ*^15^N values. Stable isotope ratios were expressed as delta values (*δ*) in per mil units (‰), which represent the ratios of heavy to light isotopes within a sample (R_sample_), relative to the ratio in an international standard (R_standard_) as:

$$\delta =\left(\left(\frac{R_{\mathrm{sample}}}{R_{\mathrm{standard}}}\right)-1\;\right)\times \;1000$$

### 2.4. Sulphur Analysis

A subset of 36 skin samples from mature individuals was processed for sulphur isotope analysis at IsoTrace Limited, Dunedin. Samples were analysed using the Carlo Erba NC 2500 elemental analyser coupled to a Europa Hydra isotope ratio mass spectrometer. Stable isotope values were normalised against international standards of Vienna PDB, AIR and Canyon Diablo Troilite for carbon, nitrogen, and sulphur, respectively. Two international reference materials comprising USGS40 mixed with IAEA-S1 (carbon = −26.39‰, nitrogen = −4.52‰, sulphur = −0.30‰), and USGS-41 mixed with IAEA-S2 (carbon = 36.55‰, nitrogen = 47.55‰, sulphur = 22.62‰) used for data normalisation in a three-point system. Replicate analysis of the keratin internal working laboratory standard was used to determine machine drift, and precision of *ẟ*^13^C (0.08‰), *ẟ*^15^N (0.04‰) and *ẟ*^34^S (0.16‰) was assessed from replicates positioned every ten samples. 

### 2.5. Correction Equations

Lipids are depleted in ^13^C relative to ^12^C compared to proteins. The lipid content of ecological samples therefore affects *δ*^13^C values [65]. Lipids are thus either removed from the sample before carbon stable isotope analysis, e.g., [17,66,67,68] or a lipid correction equation is applied to samples with C:N mass ratios > 3.5 to correct for the lipid-affected *δ*^13^C values [69,70,71]. There is disagreement within published literature regarding the suitability of lipid correction equations being extrapolated to different species for isotopic studies [72]. Therefore, lipids were extracted from a sub-set of ten *G. m. edwardii* skin samples (Supplementary Material Table S2) to check the validity of using published lipid correction equations [69,73,74,75]. Samples were selected from one location only (FWS) based on, (1) extreme carbon and nitrogen isotope values in comparison to the rest of the dataset and, (2) a wide range of C:N mass ratios. Selected samples had C:N mass ratios ranging from 3.27–4.48 and C:N atomic ratios ranging from 3.81–5.23. The lipid correction equation, which was based on a bootstrapping approach using 74 samples of odontocetes, including *G. m. edwardii* from Peters et al. [75], was found to be the best fit for the data. The lipid correction equation:

*δ*^13^C_corrected_ = 0.5301486 × *δ*^13^C − 7.322335

was applied to *δ*^13^C values for samples with a C:N mass ratio over 3.5. Bulk isotope uncorrected *δ*^13^C values were used when C:N mass ratios were <3.5. As lipid extraction can affect nitrogen and sulphur isotope values [76], non-lipid extracted bulk samples were analysed to generate *ẟ*^15^N and *ẟ*^34^S values. Additionally, to account for changing carbon dioxide levels in the ocean due to anthropogenic activity [77], commonly referred to as the Suess effect, a correction equation of −0.022% y^−1^ [78] was applied to all *δ*^13^C values to the baseline of our most recent sample set collected in 2017. 

### 2.6. Statistical Analysis

Following testing assumptions of normality using Shapiro–Wilk tests, Kruskal–Wallis tests were used in the R package “rstatix” [79] to compare differences in mean ($\bar{x}$) *δ*^13^C, *δ*^15^N and *δ*^34^S values both within and among groups defined as: sex, reproductive group, stranding location, stranding event, and stranding year. For *δ*^13^C and *δ*^15^N values, these were also compared among maturity status, which was not an option for *δ*^34^S as we only had *δ*^34^S values for mature animals. Where significant differences occurred, pairwise data were compared using Wilcoxon tests to determine differences between specific groups, e.g., [17,80]. Spearman’s correlation coefficient was used to determine if any relationship occurred between body length or age and *δ*^13^C, *δ*^15^N and *δ*^34^S values, respectively. The relationship between *δ*^13^C, *δ*^15^N, and *δ*^34^S values and a suite of predictive variables was investigated using generalised additive models (GAMs) [81] using the R package “mgcv” [82]. Predictive variables were sex, body length, maturity status (only for *δ*^13^C and *δ*^15^N values), stranding location, stranding event and stranding year. Body length was fitted as a continuous variable, whereas sex, maturity status, stranding location, stranding event and stranding year were fitted as factors. As body length and age were highly correlated (Spearman rank, rho = 0.85, *p* ≤ 0.01), and age was not available for all individuals, body length (*n =* 125) was included in GAM models as a proxy rather than age itself (*n =* 108). Models were built with Gaussian distribution with gamma set to 1.4 to prevent overfitting [83] with all possible combinations of variables. Akaike’s information criterion adjusted for small sample size (AICc; [84]) was using the R package “qpcR” [85] to select the best fitting model. Interactions for the five top-ranked models were also tested. Final models were checked for normality and obvious patterns in the residuals. Niche partitioning was investigated using Bayesian inference using the R packages “SIBER” [27] and “ggplot2” [86] with ellipses calculated at the 0.40 and 0.95 α level. 

Niche regions (NR) were presented in three-dimensions (‰^3^) using *δ*^13^C, *δ*^15^N and *δ*^34^S data using the R packages “scatterplot3d” [87] and “nicheROVER” [88]. Volume of ellipses was set at the 0.40 α level (NR_40_, e.g., [10]). Data were split into groups based on ontogenetic variation and stranding event to calculate pairwise isotopic niche overlap. For ontogenetic variation, data were classified as mature males, mature females, and pregnant/lactating females due to data availability. Published methods were followed [89], replacing “Species” with “Group”, whereby pairwise niche overlap was defined as the probability (%) of an individual from one group being found within the NR_40_ of another group. Data were presented as a pairwise grid of one-dimensional isotopic density distributions, two-dimensional pairwise isotopic scatter plots and two-dimensional NR_40_ ellipses of five random NR_40_ estimates. Overlap probability was calculated at the 95% level using a Bayesian approach with 10,000 iterations and reported as mean posterior overlap, e.g., [10].

The relationship between number of *G. m. edwardii* stranded and triple isotope niche size was examined through Pearson’s correlation analysis both with and without FWS2009 data included. The FWS2009 stranding event appeared anomalous as it had a much larger niche size for the number of animals stranded compared to all other events, and did not fit the trend of the other stranding events. Finally, isotopic range of *δ*^13^C using the highest and lowest values were calculated using the formula:

$${∆}_{y}=\delta^{13}C_{ymax}-\delta^{13}C_{ymin}$$

where y = sample size [90]. Isotopic ranges of *δ*^15^N and *δ*^34^S were calculated in the same way at the level of (1) the entire dataset, and (2) each stranding event.

All data analysis was completed in R version 4.0.5 [91].

## 3. Results

Lipid corrections were performed on *δ*^13^C values from 71 (57%) samples, whilst 54 samples (43%) were not lipid-corrected (Supplementary Material Table S3). Following *δ*^13^C corrections for lipid content and Suess effects, *δ*^13^C data were not normally distributed (Shapiro–Wilk, W = 0.96, *p =* 0.001). Overall, neither *δ*^15^N values (Shapiro–Wilk, W = 0.83, *p* ≤ 0.05) nor *δ*^34^S values (Shapiro–Wilk, W = 0.94, *p =* 0.03) were normally distributed.

### 3.1. Ontogenetic Variation in δ^13^C, δ^15^N and δ^34^S Values

**Figure 2:**
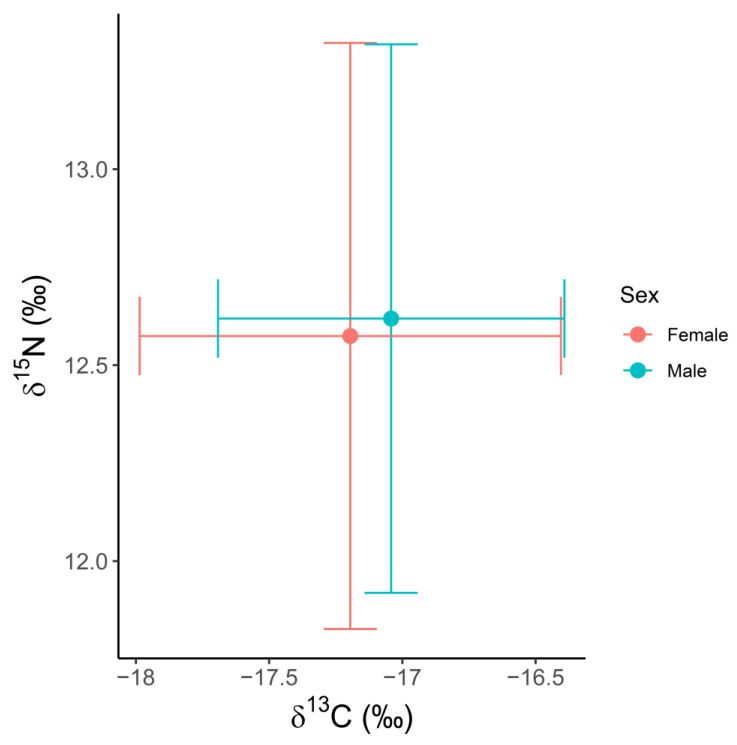
Carbon and nitrogen (*δ*^13^C and *δ*^15^N) stable isotope biplot from skin samples of male (*n* = 57) and female (*n* = 68) long-finned pilot whales (*Globicephala melas edwardii*) stranded on the New Zealand coast between 2009 and 2017.

**Figure 3:**
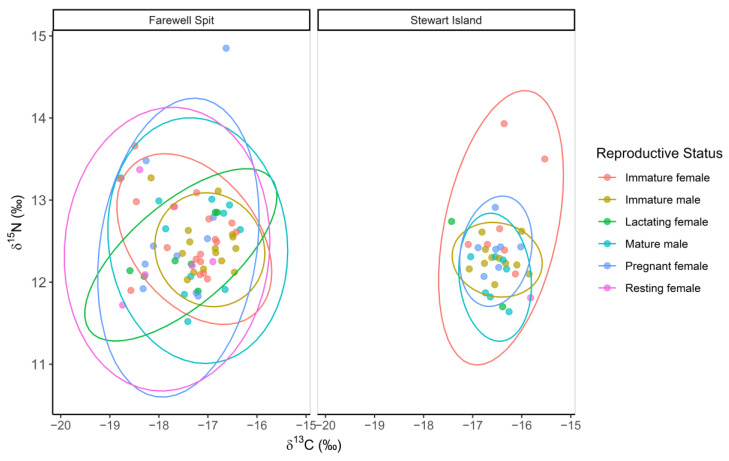
Isotopic niche overlap of carbon and nitrogen (*δ*^13^C and *δ*^15^N) isotopic values of long-finned pilot whales (*Globicephala melas edwardii*) with immature female (*n* = 25), immature male (*n* = 26), lactating female (*n* = 9), mature male (*n* = 18), pregnant (*n* = 17) and resting (*n* = 7) females presented by stranding location on the New Zealand coast, 2009–2017. Ellipses represent 95% of data.

The mean *δ*^15^N value was 12.59 ± 0.72‰ (Table 2), whilst the mean *δ*^13^C value was −17.12 ± 0.73‰ (*n* = 125). No significant correlations were found between *δ*^13^C values and body length (Spearman rank, rho = −0.06, *p =* 0.54) nor age (Spearman rank, rho = −0.12, *p =* 0.22), respectively. Futher, no significant differences were found in the *δ*^13^C values between males (−17.04 ± 0.65‰, *n* = 57) and females (−17.20 ± 0.79‰, *n* = 68; Kruskal–Wallis, ts = 0.98, *p =* 0.32, Figure 2), between immature (−17.00 ± 0.70‰, *n* = 56) and mature (−17.23 ± 0.74‰, *n* = 69) individuals (Kruskal–Wallis, ts = 2.89, *p* = 0.09) or among reproductive groups (immature males: −16.79 ± 0.81‰, *n* = 26; mature males: −17.00 ± 0.69‰, *n* = 18; immature females −17.12 ± 0.81‰, *n* = 25; pregnant females: −17.13 ± 0.73‰, *n* = 17; lactating females: −17.40 ± 0.71‰, *n* = 9; resting females: −17.59 ± 1.01‰, *n* = 7; Kruskal–Wallis, ts = 9.06, *p =* 0.11, Table 2, Figure 3). 

Similarly, no significant correlations were found between *δ*^15^N and body length (Spearman rank, rho = −0.08, *p =* 0.36) nor age (Spearman rank, rho = −0.09, *p =* 0.37), respectively. No differences in the *δ*^15^N values between males (12.62 ± 0.70‰, *n* = 58) and females (12.57 ± 0.75‰, *n* = 67, Kruskal–Wallis, ts = 0.41, *p =* 0.52), between immature (12.59 ± 0.58‰, *n* = 56) and mature individuals (12.60 ± 0.82‰, *n* = 69, Kruskal–Wallis, ts = 0.53, *p* = 0.47) or among reproductive groups (immature males: 12.38 ± 0.30‰, *n* = 26; mature males: 12.34 ± 0.53‰, *n* = 18; immature females 12.64 ± 0.53‰, *n* = 25; pregnant females: 12.53 ± 0.72‰, *n* = 17; lactating females: 12.30 ± 0.42‰, *n* = 9; resting females: 12.34 ± 0.60‰, *n* = 7; Kruskal–Wallis, ts = 6.14, *p* = 0.29) were detected (Table 2). 

The mean *δ*^34^S value was 21.42 ± 0.91‰ for the pooled dataset (*n* = 36). Sulphur isotope values did not differ significantly between sex (males 21.14 ± 0.99, *n* = 13; females 21.58 ± 0.83, *n* = 23; Kruskal–Wallis, ts = 1.87, *p* = 0.17, Table 3). Similarly, no significant correlations were found between *δ*^34^S and age (Spearman rank, rho = −0.20, *p =* 0.27) nor body length (Spearman rank, rho = −0.21, *p =* 0.22).

### 3.2. Spatial and Temporal Variation in δ^13^C, δ^15^N and δ^34^S Values

**Figure 4:**
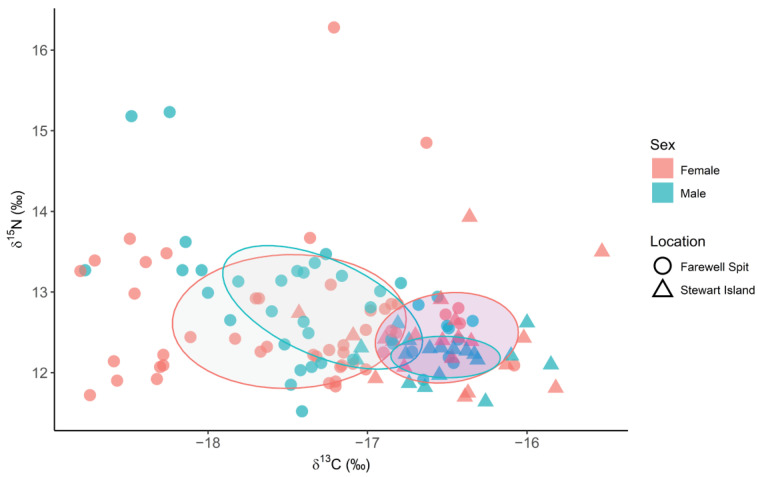
Long-finned pilot whales (*Globicephala melas edwardii*) isotopic niche overlap of carbon and nitrogen (*δ*^13^C and *δ*^15^N) values between males (*n* = 40) and females (*n* = 47) stranded at Farewell Spit, and males (*n* = 18) and females (*n* = 20) stranded at Stewart Island between 2009 and 2017. Stewart Island is represented as triangles and purple filled ellipses, and Farewell Spit as circles and grey filled ellipses, males are indicated in green and females in peach. Ellipses represent 40% of the data.

Overall, individuals that stranded at FWS (*n* = 87) had significantly lower *δ*^13^C and higher *δ*^15^N values (*δ*^13^C—17.39 ± 0.68‰, *δ*^15^N 12.71 ± 0.79‰) compared to those stranded at SI (*δ*^13^C—16.51 ± 0.39‰, *n* = 38; Kruskal–Wallis, ts = 45.6, *p* ≤ 0.01; *δ*^15^N 12.32 ± 0.44‰, Kruskal–Wallis, ts = 8.43, *p* ≤ 0.01, Figure 4). Total niche area (TA) and corrected standard ellipse areas (SEA_C_) were larger for females at both FWS (female TA = 7.64, SEA_C_ = 1.89, *n* = 47; male TA = 4.72, SEA_C_ = 1.25, *n* = 40) and SI (female TA = 2.74, SEA_C_ = 0.79, *n* = 20; male TA = 0.83, SEA_C_ = 0.28, *n* = 18). The TA and SEAc values were larger at FWS than SI for both males and females, respectively. The TA was largest for pregnant females at FWS (TA = 3.14, SEA_C_ = 1.96, *n* = 10), and smallest for mature males at SI (TA = 0.30, SEA_C_ = 0.27, *n* = 7; Supplementary Material Table S4).

Differences in *δ*^13^C were recorded between stranding events (Kruskal–Wallis, ts = 89.7, *p* =< 0.01), with Wilcoxon tests describing four pairs as not significantly different: FWS2014, FWS2017 (*p* = 0.40); and FWS2009, SI2010 (*p* = 1); FWS2009, SI2011 (*p* = 0.25) and SI2010, SI2011 (*p* = 0.25). Mean *δ*^13^C values were lowest in FWS2017 stranded individuals ($\bar{x}\;$ = −18.04 ± 0.52‰, *n* = 20), whereas the highest mean *δ*^13^C values were observed in those stranded at FWS2009 ($\bar{x}$ = −16.65 ± 0.31‰, *n* = 20). Nitrogen isotope values differed among stranding events (Kruskal–Wallis, ts = 57.1, *p* ≤ 0.01), with higher *δ*^15^N values recorded in individuals from FWS2017 (*n* = 20) than any other stranding event. Nitrogen isotope values were also lower at the FWS2014 stranding event (*n* = 27) than any other FWS stranding event. 

**Figure 5:**
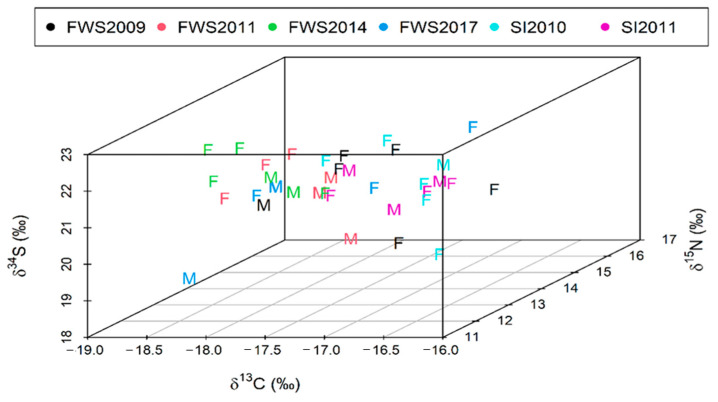
Carbon, nitrogen, and sulphur *(δ^1^*^3^C*, δ*^15^N and *δ^3^*^4^S*)* stable isotope triplot of long-finned pilot whale (*Globicephala melas edwardii*) skin samples. Males are represented by “M” and females by “F”. Data are presented by stranding event as indicated by colour in the legen3.3. GAM Analysis.

Sulphur isotope values did not differ significantly between stranding events (Kruskal–Wallis, ts = 9.24, *p* = 0.10) nor stranding location (FWS: 21.33 ± 0.95‰, *n* = 32; SI: 21.61 ± 0.82‰, *n* = 18; Kruskal–Wallis, ts = 0.65, *p* = 0.42; Figure 5).

The top three GAMs for *δ*^15^N retained only stranding event, location, and year. The top model retained only stranding event as a covariate, explaining 45% of the deviance (Table 4). For *δ*^13^C values, the top two best-fit models retained maturity status, location, year and stranding event as covariates and explained 69% of the deviance. Sex stranding event was also retained as a covariate in the top three GAMs fitted for *δ*^13^C data (Table 4). Whilst body length was also fitted to GAMs, this was not retained in the top-ranked models. The top-ranked GAM for *δ*^34^S retained only sex as a covariate. Stranding location and year were also retained, respectively, as covariates in the top three models (Table 4). However, the deviation explained was less than 10% for all models (Table 4), indicating that the included predictor variables did not explain the data well.

### 3.3. Triple Isotope Niche Regions

**Figure 6:**
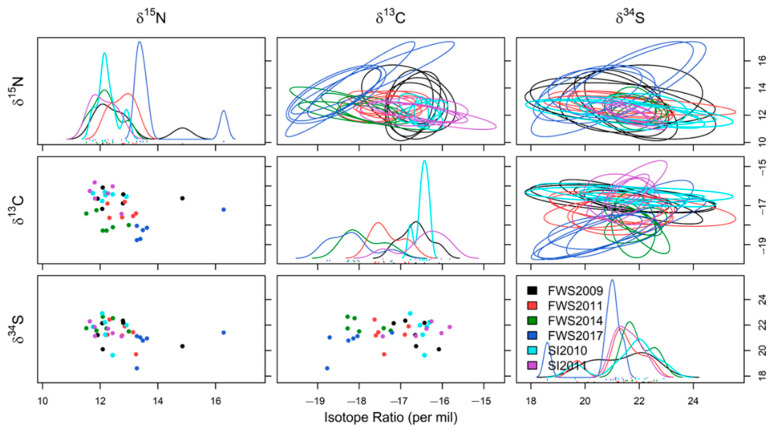
Two-dimensional scatterplots, one-dimensional density plots and two-dimensional 95% niche overlap ellipses of five random skin samples of carbon, nitrogen, and sulphur isotopes (*δ*^13^C, *δ*^15^N and *δ*^34^S) of long-finned pilot whales (*Globicephala melas edwardii*) from each of six stranding events on the New Zealand coast, 2009–2017. In the sample identifiers, FWS = Farewell Spit, SI = Stewart Island.

Triple isotope niche regions at the α = 40 level (NR_40_) were calculated by ontogenetic variation and stranding event. Pairwise comparisons showed the NR_40_ overlaps of individuals from differing ontogenetic groups (Table 5a). Females had the most unique isotopic niche space, with only a 48% chance any resting females would be found in the NR_40_ of mature males but a 75% chance they would be found in the NR_40_ of pregnant/lactating females (Table 5a). However, there was a high degree of probability that both mature males (82%) or pregnant/lactating females (91%) would be found within the NR_40_ of all females. Likewise, mean niche size was much larger for all females (mean ± SE = 53.58 ± 13.82‰^3^) than either pregnant/lactating females (33.56 ± 11.24‰^3^) or males (20.72 ± 7.18‰^3^). Mean niche size was similar across several stranding events; FWS2011 (6.62 ± 3.60‰^3^), FWS2014 (4.32 ± 2.27‰^3^), SI2011 (4.11 ± 2.21‰^3^) and SI2010 (3.78 ± 2.04‰^3^). The combined niche width of individuals stranded at FWS2009 (17.62 ± 9.42‰^3^) and FWS2017 (15.52 ± 8.25‰^3^) were much larger than those of all other stranding events (Figure 6).

There was a 59% chance of an individual from SI2011 being found in the NR_40_ of FWS2009, the highest probability recorded. However, there was only a 1% chance of an individual from FWS2017 being found within the NR_40_ of SI2010. Individuals stranded at FWS had a 0–36% chance of being found in the NR_40_ of individuals stranded at SI, whereas there was a much higher chance (0–75%) of an individual from SI being found in the NR_40_ of an individual stranded at FWS. Several pairs were considered to have low probability of NR_40_ overlap (<10%), with individuals from FWS2017 seemingly the least likely to be detected within the NR_40_ of any other stranding event (Table 5b). The NR_40_ overlap appeared high both between stranding events occurring at the same site (e.g., SI2010 and SI2011) and those that were temporally close (e.g., FWS2009 and SI2010 which occurred only three months apart, Table 5b). 

No significant correlation between stranding group size and niche size (correlation = 0.55, *p* = 0.26) was detected. However, when the FWS2009 stranding event was removed from the dataset, a significant positive correlation was revealed between the number of animals involved in the stranding event and the niche width (correlation = 0.92, *p* = 0.03). Finally, isotopic range was found to be similar between *δ*^13^C (3.27‰), *δ*^15^N (4.75‰) and *δ*^34^S (4.30‰) values for the entire pooled dataset (Table 6). The smallest range of *δ*^13^C values were found at the SI2010 stranding event (0.76‰) along with the largest range of *δ*^34^S values (3.27‰). In contrast, the largest range of *δ*^13^C values were recorded at the SI2011 stranding event (1.90‰) and the smallest range of *δ*^34^S values (1.15‰) were recorded at FWS2014. Finally, the largest range of *δ*^15^N values were recorded at the FWS2017 (3.85‰) stranding event, whereas the smallest range was at the FWS2011 stranding (1.20‰). 

## 4. Discussion

Intraspecific variation in isotopic values has been explored in multiple cetacean species [1]. Here, we analysed ontogenetic and spatiotemporal effects on the isotopic niche of a single cetacean species, *G. m. edwardii*. Overall, isotopic data from this study were characterised by a high level of overlap between the 125 individuals analysed for *δ*^13^C and *δ*^15^N and the 36 individuals analysed for *δ*^34^S. Significant differences were found in both *δ*^13^C and *δ*^15^N, but not *δ*^34^S values when examined by location stranded and stranding event. No significant differences were found in *δ*^13^C, *δ*^15^N and *δ*^34^S values vs. sex, body length, age, maturity status or reproductive group.

In general, mean *δ*^15^N values for *G. m. edwardii* reported in this study ($\bar{x}=$ 12.71‰, *n* = 125) were lower than mean values reported for other cetacea in New Zealand waters around the same time period, e.g., teuthophagus common dolphins *Delphinus delphis* (female $\bar{x}=$ 14.88‰, *n* = 33; male $\bar{x}=$ 14.81‰, *n* = 23; [92]) and male sperm whales *Physeter macrocephalus* ($\bar{x}=$ 15.6‰, *n* = 37; [93]). However, mean *δ*^15^N values were still higher than other New Zealand marine mammals with diets that are more focused on copepods and krill, such as blue whales *Balaenoptera musculus* sp. ($\bar{x}=$ 11.1‰, *n* = 8; [94]) and southern right whales *Eubalaena australis* ($\bar{x}=$ 8.09‰, *n* = 18; [95]). Lower *δ*^15^N values were also recorded in *G. m. melas* in the Mediterranean in comparison to other teuthophagus odontocetes such as *P. macrocephalus* and Risso’s dolphins *Grampus griseus* [35]. This could be indicative of offshore feeding [96,97,98]. Indeed, *δ*^15^N values reported in this study were consistent with those of other *G. melas* populations globally [37,47,99,100,101]. Similarly, *δ*^13^C values recorded here were comparable to those measured in northern hemisphere *G. m. melas* populations [47,102,103]. 

Sulphur isotopes can provide useful information on foraging prey source pathways [20,21], *G. m. edwardii δ*^34^S values from this study were similar to those reported in *G. m. melas* in the Mediterranean [102]. High *δ*^34^S ($\bar{x}=$ 21.52‰, *n* = 36) values indicated a large contribution to diet from marine sulphate, indicating marine foraging pathways [20,104,105]. The combination of low *δ*^13^C values with high *δ*^34^S values observed in this study has previously been described as typical of oceanic feeding behaviour [106], corroborating that *G. melas* is primarily an oceanic species [34,107,108]. 

### 4.1. Ontogenetic Variation in Isotope Values

No observable differences in isotopic niche among the different ontogenetic groups were detected in this study, aligning with observed isotopic homogeneity of *G. m. melas* in the Strait of Gibraltar [37]. Whilst sex differences in resource-use have been reported in other cetacean species including bottlenose dolphins *T. truncatus* [109], this has not been recorded in *G. melas* previously. Furthermore, higher cadmium levels have been reported in female *G. m. edwardii* from New Zealand waters than in males [110]. Higher cadmium load in females could signify a greater reliance on cephalopod prey [111], as cephalopods are known to accumulate cadmium in their tissues [112]. Females had a larger TA than males when considering only *δ*^13^C and *δ*^15^N values and were less likely to be found in the triple isotope niche of males (56%) than the other way around (79%, Table 5a). Yet, no differences were detected in mean *δ*^13^C, *δ*^15^N or *δ*^34^S values between males and females. Whilst sex was retained as a predictor in the top-ranked GAM for *δ*^34^S (Table 4), the deviance explained was very low (6%), indicating that there are likely other factors that determine *δ*^34^S values. 

Like many cetacean species, *G. m. edwardii* displays sexual dimorphism with males being larger than their female counterparts [60,113]. It is possible that increased overall body size, rather than sex, could be driving the small isotopic niche differences reported here. However, body length was not retained as a predictor in the top-ranked models for *δ*^13^C, *δ*^15^N or *δ*^34^S (Table 4) nor significantly correlated with isotopic values. Whilst maturity status was retained as a predictor explaining *δ*^13^C variation, this was not the case for *δ*^15^N or *δ*^34^S data. Hence, this study did not reveal a link between consumption of prey from higher trophic levels and body length. Similarly, no relationship was evident between stable isotope values and body length in *P. macrocephalus* [93,114], or *δ*^34^S, sex and body size in *T. truncatus* [104]. 

An increased reliance on higher trophic levels with increased body length has been reported in weaned striped dolphins *Stenella coeruleoalba* [42,115], whilst studies of *P. macrocephalus*, Commerson’s dolphins *Cephalorhynchus commersonii commersonii*, common dolphins *D. capensis* and *T. truncatus* all reported an increase in *δ*^15^N with age [9,38,44,116]. Though no statistical relationship was apparent between isotope values and body length or age in this dataset, high *δ*^15^N values were recorded in some of the smallest and youngest pilot whales, which is consistent with reliance on lactation in young cetacea [68,117,118]. 

The effect of reproduction on stable isotope values in cetaceans has not been as well studied, but it has been suggested that energetic demands and nutrient intake of mature females can differ due to reproductive status [119,120,121]. In this study, pregnant females had the largest isotopic niche of all reproductive groups. It has been suggested that the specific stage of pregnancy could affect isotope values of humpback whales *Megaptera novaeangliae* [122], so further distinction in reproductive groups, including pregnancy stage, may be necessary to elucidate isotopic variability. Furthermore, lactating *G. m. edwardii* often had higher *δ*^15^N values than resting females, though this difference was not statistically significant. In general, older females that are no longer reproductively active may target riskier prey [123], causing a change to their isotopic niche. However, resting *G. m. edwardii* in this study were not necessarily of advanced age. Overall, isotopic homogeneity among reproductive groups could be due to trophic similarity within the population, lack of sufficient samples within each stranding event or indeed, varying stages of pregnancy. A similar lack of variation in isotope values by reproductive group has been reported in sei whales, *B. borealis* and Bryde’s whales, *B. edeni* [124]. Differences in isotopic values that do not meet the threshold for statistical significance have been previously proven ecologically significant through the use of complementary dietary analysis methods such as fatty acid analysis [125]. Accordingly, future examination of fatty acid profiles for the New Zealand *G. m. edwardii* population could shed further light on their foraging ecology.

### 4.2. Spatial and Temporal Variation in Stable Isotope Values

Spatial differences in isotopic composition within a population are well recorded in cetacea, including *G. melas* [47,100]. For example, spatial differences in *δ*^15^N values have been attributed to prey selection and trophic breadth, whilst differences in δ^13^C have been linked to feeding area (e.g., offshore or coastal) and latitude [1,8]. It was predicted that SI strandings events would have lower *δ*^13^C values compared to FWS due to the more southerly location [126], however the opposite was true (Figure 4). Furthermore, *δ*^15^N values were consistently lower in SI than FWS. The lack of significant differences in sulphur isotope values suggests that these carbon and nitrogen isotopic variances are likely due to variation in primary productivity and baseline isotope values between the two locations rather than differences in diet or food web pathways. Future studies would benefit from baseline isotopic information obtained from either: (1) sampling suspended particulate organic matter in surface waters or a sessile primary consumer; or (2) employing compound specific isotope analysis to tease out confounding baseline versus trophic level drivers of elevated *ẟ*^15^N values [127,128,129,130].

The isotopic ranges of values per stranding event for carbon, nitrogen and sulphur were much smaller than those observed in the overall dataset. Furthermore, stranding event was retained in three of the top six GAMs reported for *ẟ*^13^C and *ẟ*^15^N, indicating that stranding event was an important driver of variation for carbon and nitrogen isotopic values. Individuals involved in the SI2010 mass-stranding had the smallest niche size of all the stranding events, which indicates little inter-individual difference in prey and foraging locations for animals involved in this stranding event. However, SI2010 was also the event with the smallest number of overall individuals stranded (Table S1) which may confound the results. The widest NR_40_ was recorded at FWS2009 even though this stranding did not comprise the most animals stranded. When FWS2009 was removed from the dataset, a positive correlation was seen between niche size and the total number of *G. m. edwardii* from all other stranding events. Although long-finned pilot whales are generally believed to live in matrilineal pods [131,132], mass-stranding events of *G. m. edwardii* on the New Zealand coast have been reported to involve individuals from many different maternal lineages [133]. This wider NR_40_ and isotopic variability could therefore signify multiple groups that have previously been dispersed from each other [134], but have fused to form a “super pod” shortly prior to stranding. With little other information available, such as genetic barcoding for individuals within stranding events, it is impossible to assume the genetic or social composition of the FWS2009 stranding event. It could be that the individuals stranded in FWS2009 represented a single pod. If that were the case, a wide NR could indicate a more heterogeneous feeding strategy or utilisation of more varied resources [135]. Both a wide isotopic niche and heterogeneity of isotopic niche within a population can indicate a generalist feeding strategy, diversified diet, or a degree of individual dietary specialization [136,137,138].

The large niche size recorded in FWS2009 appeared to be driven by a larger range of *δ*^15^N values compared to other stranding events (Table 6). The individuals stranded in the FWS2017 event also recorded a large niche size, driven by both the largest range of *δ*^15^N values and second largest range of *δ*^34^S values compared to other stranding events (Table 6). This indicates that individuals in these two stranding events had a more varied diet. This could be due to ingestion of a mixture of different trophic level prey which themselves feed in a variety of benthic/pelagic, and coastal/oceanic habitats. Isotopic density plots for both FWS2011 and SI2010 (Figure 6) also had lower *δ*^34^S values, suggesting an inshore or benthic component to feeding prior to these stranding events [20,139]. Globally, *G. melas* have been recorded as having dietary plasticity, displaying behavioural changes by following prey that have migrated due to changes in oceanic currents and water temperatures and adapting their diet to locally available prey [49,140]. Indeed, observations of a single captive *G. m. melas* showed a preference shift to the more abundant prey when prey proportions were varied [141]. Stomach content studies of *G. m. edwardii* from New Zealand waters suggest a large dietary reliance on arrow squid *Nototodarus* spp. [51,52,53]. 

It is difficult to ascertain whether changes such as a widening NR_40_ are indicative of a temporal niche change since the data in this study only span a few years. Whilst δ^15^N values were highest in 2009 they were also high in 2017, suggesting there is not a linear temporal pattern in *δ*^15^N values. However, a temporal decline in *δ*^13^C values at FWS was noted between 2009 and 2017 (Figure 6), echoing similar findings from marine predators such as tuna (*Tunnus albacares*, *T. obesus* and *T. alalunga*; [142]) and *D. delphis* [92] across the Pacific Ocean in recent years. Whilst seasonal differences in prey have been recorded in *G. m. melas* population in the northern hemisphere [37,48], data in this study are exclusively from mass-strandings that occurred during the austral summer (November to February) in New Zealand [50], preventing seasonal comparisons.

Resource partitioning of socially and spatially distinct groups has been noted in other cetacea [115,143]. Although stranding records and sightings data show that *G. m. edwardii* strand all around New Zealand, only two stranding hotspot locations [50] were explored here. Despite the geographic separation of SI and FWS (800 km apart) there was little isotopic variability between stranding events at the two locations when strandings occurred within the same year. In the absence of tracking, genetic, or migratory data, it is not known whether any surviving members of the FWS2009 stranded pod were involved in the stranding event three months later at SI. 

Population homogeneity has been recorded in northern hemisphere *G. m. melas* populations [144], suggesting that individuals in the same pods may feed in similar environments. As stranding event appeared to be the most prominent predictor of niche, a degree of individual/group specialisation [37] or cooperative foraging may exist, as has been observed in other odontocetes [145]. Multiple feeding techniques have been observed in *G. melas* populations, including both shallow and deep foraging dives [146,147] and nocturnal [148,149] and suction feeding [150] in captive animals. Satellite tagging of the closely related short-finned pilot whales *G. macrorhynchus* in the northeastern Atlantic revealed that individuals may be able to adapt foraging states and behaviour per dive in response to immediate physiological and environmental constraints [151]. However, it is not clear what foraging strategy *G. m. edwardii* utilise in New Zealand waters due to a lack of tagging, video, or distribution data for this species.

## 5. Conclusions

This study was the first to investigate isotopic variation of *G. m. edwardii* in New Zealand waters. Overall, spatiotemporal variation appeared to have a greater effect on isotopic values than ontogenetic variation, with significant differences in *δ*^13^C and *δ*^15^N values detected between stranding location and event. Whilst *δ*^34^S values did not directly relate to ontogenetic or spatiotemporal factors, incorporating sulphur isotope data improved isotopic niche calculations and provided insight into drivers of other isotopic differences. In particular, *δ*^34^S values determined possible drivers of isotopic niche differences between stranding events, which were not easily identified using just *δ*^13^C and *δ*^15^N values. Finally, our study showed the benefits of long-term tissue archiving when supported by robust life history datasets. Further sampling of *G. m. edwardii* and their associated prey from additional locations over multiple seasons would improve understanding of spatial and seasonal niche changes for *G. m. edwardii*. In addition, satellite tagging of *G. m. edwardii* individuals would provide missing information about their movements, foraging ranges, and habitats. 

## Figures and Tables

**Table 1:** Ontogenetic characteristics of long-finned pilot whales (*Globicephala melas edwardii*) sampled for stable isotope analysis from mass-strandings on the New Zealand coast, 2009–2017. Unknown refers to individuals where reproductive group was unable to be determined from reproductive organs, but maturity status was instead classified from body length.

Ontogenetic Status	*n*	Body Length Range (cm)	Age Range (Years)
*Maturity status*			
Immature	56	168–482	0–13
Mature	69	364–595	6–33
*Reproductive group*			
Immature male	26	255–482	1–13
Mature male	18	467–581	14–31
Immature female	25	168–375	0–8
Pregnant female	17	364–461	6–33
Lactating female	9	380–446	7–30
Resting female	7	397–453	11–30
Unknown	23	194–595	5–32

**Table 2:** Range, mean and standard deviations (±1 SD) of carbon and nitrogen (*δ*^13^C and *δ*^15^N) values of long-finned pilot whales (*Globicephala melas edwardii*) stranded on the New Zealand coast, 2009–2017, presented by sexual maturity status and reproductive group. Unknown refers to individuals where reproductive group was unable to be determined from reproductive organs, but maturity status was instead classified from body length.

		*δ*^13^C (‰)			*δ*^15^N (‰)		
	*n*	Range	Mean	SD	Range	Mean	SD
All	125	−18.80 to −15.53	−17.12	0.73	11.52 to 16.28	12.59	0.72
*Maturity status*							
Immature	56	−18.80 to −15.53	−17.00	0.70	11.90 to 15.23	12.59	0.58
Mature	69	−18.77 to −15.82	−17.23	0.74	11.52 to 16.28	12.60	0.82
*Reproductive group*							
Immature male	26	−18.16 to −16.26	−16.79	0.52	11.97 to 13.27	12.38	0.30
Mature male	18	−18.77 to −16.26	−17.00	0.69	11.52 to 13.27	12.34	0.53
Immature female	25	−18.80 to −15.53	−17.12	0.81	11.90 to 13.93	12.64	0.53
Pregnant female	17	−18.32 to −16.02	−17.13	0.73	11.83 to 14.85	12.53	0.72
Lactating female	9	−18.59 to −16.39	−17.40	0.71	11.70 to 12.85	12.30	0.42
Resting female	7	−18.74 to −15.82	−17.59	1.01	11.72 to 13.37	12.34	0.60
Unknown	23	−18.62 to −15.99	−17.26	0.68	11.75 to 16.28	13.23	1.10

**Table 3:** Mean and standard deviations (±1 SD) of carbon, nitrogen, and sulphur (*δ*^13^C, *δ*^15^N and *δ*^34^S) values of a subset of 36 mature long-finned pilot whales (*Globicephala melas edwardii*) stranded on the New Zealand coast (2009–2017), presented by sex and reproductive group.

		*δ*^13^C (‰)		*δ*^15^N (‰)		*δ*^34^S (‰)	
	*n*	Mean	SD	Mean	SD	Mean	SD
Male	13	−17.32	0.81	12.59	0.72	21.14	0.99
Female	23	−17.06	0.82	12.70	1.04	21.58	0.83
Pregnant/Lactating female	14	−17.12	0.73	12.68	0.75	21.56	0.90
All	36	−17.14	0.78	12.66	0.93	21.42	0.91

**Table 4:** Summary statistics for the top three generalised additive models (GAMs) selected based on Akaike Information Criterion corrected for small samples sizes (AICc) of long-finned pilot whale (*Globicephala melas edwardii*) skin samples, presented by carbon, nitrogen and sulphur (*δ*^15^N, *δ*^13^C and *δ*^34^S) values. LL: log-likelihood; % DE: % deviance explained; ΔAICc: difference in Akaike’s information criterion (AIC_C_) of the current and top-ranked model; wAICc = AIC_C_ weight. Significant variables are highlighted in bold.

Model	R^2^	LL	% DE	∆AICc	wAICc
*δ*^15^N					
**Stranding event**	0.431	1.000	45.40	-	0.145
Location + **Stranding event**	0.431	0.885	45.40	0.250	0.128
**Year** + Location	0.425	0.553	44.90	1.190	0.080
*δ*^13^C					
**Maturity** + **Stranding event**	0.679	1.000	69.40	-	0.119
**Maturity** + **Year** + **Location**	0.679	1.000	69.40	-	0.119
Sex + **Maturity** + **Year** + **Location**	0.680	0.87	69.80	0.284	0.103
*δ*^34^S					
Sex	0.030	1.000	5.59	-	0.211
Year	0.020	0.885	4.77	0.250	0.186
Location	0.030	0.486	8.85	1.440	0.102

**Table 5:** Confusion matrices of triple isotope (*δ*^13^C, *δ*^15^N and *δ*^34^S) niche overlap at the 95% confidence level of mature long-finned pilot whales (*Globicephala melas edwardii*) processed from stranding events on the New Zealand coast between 2009 and 2017. Values are the chances (%) that an individual from the group on the left-hand column would be found within isotope niche of any of the other groups in its row. Data presented by (**a**) maturity status and (**b**) stranding event.

**(a)**							
		**Mature Male**		**Mature Female**		**Pregnant/Lactating Female**	
Male				82.14		75.73	
Female		48.41				74.90	
Pregnant/Lactating female		57.30		91.45			
**(b)**							
	**FWS2009**	**FWS2011**	**FWS2014**		**FWS2017**	**SI2010**	**SI2011**
FWS2009		21.05	2.65		0.86	25.02	21.11
FWS2011	36.13		17.57		4.07	5.81	27.04
FWS2014	7.94	26.35			9.61	0.38	8.16
FWS2017	1.49	3.51	2.48			0.00	0.75
SI2010	74.78	20.34	1.09		0.01		41.57
SI2011	58.80	29.13	7.26		0.76	37.41	

**Table 6:** Isotopic range expressed as a percentage of carbon, nitrogen, and sulphur (*δ*^13^C, *δ*^15^N and *δ*^34^S) values of long-finned pilot whales (*Globicephala melas edwardii*) sampled from mass-stranding events on the New Zealand coast, 2009–2017. Data are presented by overall dataset, and by each stranding event: FWS = Farewell Spit, SI = Stewart Island.

Isotope Range (‰)	Overall	FWS2009	FWS2011	FWS2014	FWS2017	SI2010	SI2011
*n*	125	20	20	27	20	19	19
*δ*^13^C	3.27	1.15	0.96	1.64	1.58	0.76	1.90
*δ* ^15^N	4.76	2.95	1.96	1.47	3.85	2.18	1.86
*n*	36	6	6	6	6	6	6
*δ*^34^S	4.30	2.24	2.72	1.15	2.80	3.27	1.18

## Data Availability

Data are available in the Supplementary Material.

## Supplementary Materials

The following are available online at https://www.mdpi.com/article/10.3390/biology11101414/s1, Table S1: Summary of long-finned pilot whale (*Globicephala melas edwardii*) skin samples used for carbon and nitrogen stable isotope analysis (*n* = 125) by year and location of stranding event on the New Zealand coast. The number of animals stranded at each event (No. stranded), and the total number included in isotope analysis (No. sampled) are reported. Sex and reproductive group are taken from the same *G. m. edwardii* population [60,61]; Table S2: Bulk carbon and nitrogen and lipid-extracted carbon stable isotope values of the subset of 10 long-finned pilot whales (*Globicephala melas edwardii*) chosen for lipid extraction. “Difference” is the difference between lipid extracted and bulk non-lipid extracted ẟ^13^C values; Table S3: Range of carbon, nitrogen, and sulphur (*δ*^13^C, *δ*^15^N and *δ*^34^S) including lipid-corrected and Suess-corrected *δ*^13^C values and C:N mass ratios of long-finned pilot whales (*Globicephala melas edwardii*). Where duplicate samples were performed, the mean is given. Lab 1 = Environmental and Ecological Stable Isotope Analytical Facility, National Institute of Water and Atmospheric Research (Taihoro Nukurangi), Lab 2 = IsoTrace Limited; Table S4: Isotopic niche total area (TA), standard ellipse area (SEA) and standard ellipse area corrected (SEAc) of *δ*^13^C and *δ*^15^N values for different reproductive groups of long-finned pilot whales (*Globicephala melas**. edwardii*). Data are presented by location of stranding of *G. m. edwardii*. Figure S1: Comparison of normalised *δ*^13^C values of long-finned pilot whale (*Globicephala melas edwardii*) Lab 1 (Environmental and Ecological Stable Isotope Analytical Facility, National Institute of Water and Atmospheric Research; Taihoro Nukurangi), Lab 2 (IsoTrace Limited); Figure S2: Comparison of normalised *δ*^15^N values of long-finned pilot whale ( *Globicephala melas edwardii*) from Lab 1 (Environmental and Ecological Stable Isotope Analytical Facility, National Institute of Water and Atmospheric Research; Taihoro Nukurangi), Lab 2 (IsoTrace Limited).
