# Supplementary material for: Isotopic Niche Analysis of Long-Finned Pilot Whales (Globicephala melas edwardii) in Aotearoa New Zealand Waters
Source: Biology (Basel). 2022 Sep 28;11(10):1414. doi: 10.3390/biology11101414 (PMC9598128; doi:10.3390/biology11101414)
Supplement: Supplementary file 1 [file biology-11-01414-s001.zip › Figure S1 and S2. Lab comparisons of C and N values.pdf]

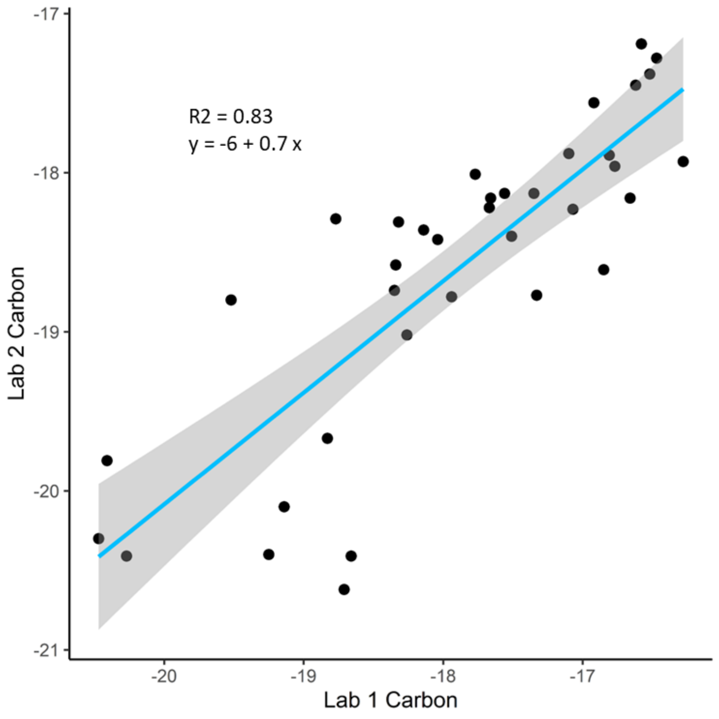

Figure S1. Comparison of normalised $\delta^{13}C$ values of long-finned pilot whale (*Globicephala melas edwardii*) Lab 1 (Environmental and Ecological Stable Isotope Analytical Facility, National Institute of Water and Atmosphere; Taihoro Nukurangi), Lab 2 (IsoTrace Limited).

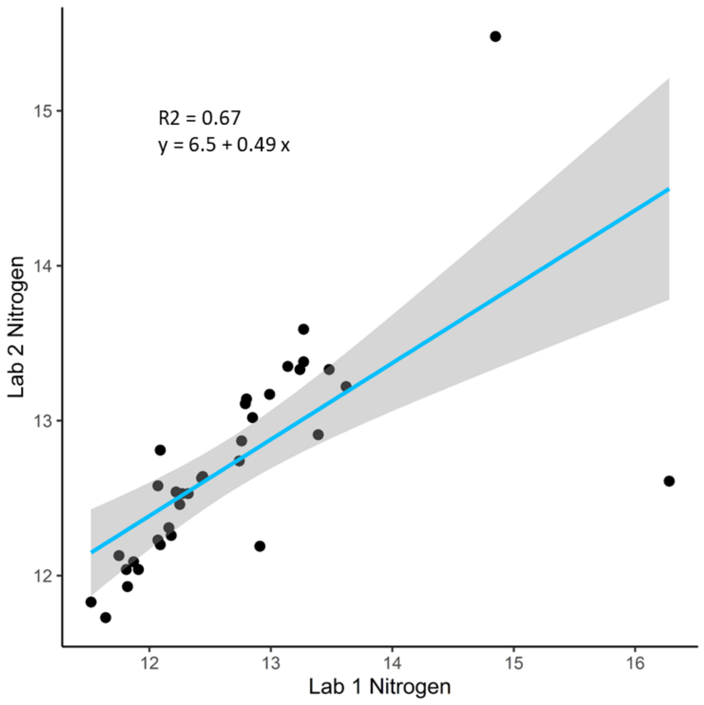

Figure S2. Comparison of normalised $\delta^{15}N$ values of long-finned pilot whale (*Globicephala melas edwardii*) from Lab 1 ((Environmental and Ecological Stable Isotope Analytical Facility, National Institute of Water and Atmosphere; Taihoro Nukurangi), Lab 2 (IsoTrace Limited).
