# Supplementary material for: Isotopic Niche Analysis of Long-Finned Pilot Whales (Globicephala melas edwardii) in Aotearoa New Zealand Waters
Source: Biology (Basel). 2022 Sep 28;11(10):1414. doi: 10.3390/biology11101414 (PMC9598128; doi:10.3390/biology11101414)
Supplement: Supplementary file 1 [file biology-11-01414-s001.zip › Table S1. Summary of samples.pdf]

**Table S1.** Summary of long-finned pilot whale (*Globicephala melas edwardii*) skin samples used for carbon and nitrogen stable isotope analysis (*n* = 125), by year and location of stranding event on the New Zealand coast. The number of animals stranded at each event (No. stranded), and the total number included in isotope analysis (No. sampled) are reported. Sex and reproductive group are taken from the same *G. m. edwardii* population [60,61].

| Date | Location | No. sampled/ No. stranded | Sex | Reproductive Group | *n* |
|---|---|---|---|---|---|
| December 2009 | Farewell Spit | 20/105 | M | Immature | 5 |
| | | | M | Mature | 5 |
| | | | F | Immature | 5 |
| | | | F | Pregnant | 2 |
| | | | F | Lactating | 0 |
| | | | F | Resting | 0 |
| | | | F | Undetermined mature | 3 |
| November 2011 | Farewell Spit | 20/65 | M | Immature | 5 |
| | | | M | Mature | 5 |
| | | | F | Immature | 4 |
| | | | F | Pregnant | 2 |
| | | | F | Lactating | 2 |
| | | | F | Resting | 2 |
| | | | F | Undetermined mature | 0 |
| January 2014 | Farewell Spit | 27/138 | M | Immature | 5 |
| | | | M | Mature | 5 |
| | | | F | Immature | 4 |
| | | | F | Pregnant | 5 |
| | | | F | Lactating | 5 |
| | | | F | Resting | 3 |
| | | | F | Undetermined mature | 0 |
| January 2017 | Farewell Spit | 20/>400 | M | Immature | 5 |
| | | | M | Mature | 5 |
| | | | F | Immature | 5 |
| | | | F | Pregnant | 1 |
| | | | F | Lactating | 0 |
| | | | F | Resting | 1 |
| | | | F | Undetermined mature | 3 |
| February 2010 | Stewart Island | 19/28 | M | Immature | 7 |
| | | | M | Mature | 2 |
| | | | F | Immature | 2 |
| | | | F | Pregnant | 5 |
| | | | F | Lactating | 0 |
| | | | F | Resting | 0 |
| | | | F | Undetermined mature | 3 |
| February 2011 | Stewart Island | 19/107 | M | Immature | 4 |
| | | | M | Mature | 5 |
| | | | F | Immature | 5 |
| | | | F | Pregnant | 2 |
| | | | F | Lactating | 2 |
| | | | F | Resting | 1 |
| | | | F | Undetermined mature | 0 |
