# Supplementary material for: Isotopic Niche Analysis of Long-Finned Pilot Whales (Globicephala melas edwardii) in Aotearoa New Zealand Waters
Source: Biology (Basel). 2022 Sep 28;11(10):1414. doi: 10.3390/biology11101414 (PMC9598128; doi:10.3390/biology11101414)
Supplement: Supplementary file 1 [file biology-11-01414-s001.zip › Table S2. Lipid extracted values.pdf]

Table S2. Bulk carbon and nitrogen and lipid-extracted carbon stable isotope values of the subset of 10 long-finned pilot whales (*Globicephala melas edwardii*) chosen for lipid extraction. "Difference" is the difference between lipid extracted and bulk non-lipid extracted $\delta^{13}C$ values.

ID	Sex	$\delta^{15}N$ (‰)	$\delta^{13}C$ (‰)	Lipid extracted $\delta^{13}C$ (‰)	Difference (‰)
Gm442	Female	11.87	-18.83	-17.87	0.95
Gm451	Male	12.16	-18.55	-17.61	0.94
Gm475	Male	12.65	-20.00	-17.99	2.01
Gm476	Male	12.99	-20.27	-17.88	2.39
Gm525	Male	13.27	-18.04	-17.95	0.09
Gm579	Female	12.42	-19.82	-17.91	1.91
Gm609	Male	13.13	-19.78	-18.37	1.41
Gm613	Male	13.62	-20.41	-17.98	2.43
Gm627	Female	12.92	-19.57	-17.94	1.63
Gm642	Female	16.28	-18.66	-18.62	0.04
Average		13.13	-19.39	-18.01	1.38
