# Supplementary material for: Isotopic Niche Analysis of Long-Finned Pilot Whales (Globicephala melas edwardii) in Aotearoa New Zealand Waters
Source: Biology (Basel). 2022 Sep 28;11(10):1414. doi: 10.3390/biology11101414 (PMC9598128; doi:10.3390/biology11101414)
Supplement: Supplementary file 1 [file biology-11-01414-s001.zip › Table S3. Isotope values.pdf]

Table S3. Range of carbon, nitrogen, and sulphur ($\delta^{13}C$, $\delta^{15}N$ and $\delta^{34}S$), including lipid-corrected and Suess-corrected $\delta^{13}C$ values and C:N mass ratios of long-finned pilot whales (*Globicephala melas edwardii*). Where duplicate samples were performed, the mean is given. Lab 1 = Environmental and Ecological Stable Isotope Analytical Facility, National Institute of Water and Atmosphere (Taihoro Nukurangi), Lab 2 = IsoTrace Limited.

	n	**Normalised C**	**Lipid corrected C**	**Number lipid corrected**	**Suess corrected C**	**Normalised N**	**C:N ratio**	**S**
**Full dataset Lab 1**	125	-20.47 to -15.72	-18.80 to -15.66	71	-18.80 to -15.53	11.52 to 16.28	3.06 to 4.48	-
**Subset Lab 1**	36	-20.47 to -16.28	-18.77 to -15.96	18	-18.57 to -15.82	11.52 to 16.28	3.08 to 4.48	-
**Subset Lab 2**	36	-20.62 to -17.19	-	-	-	11.73 to 15.48	3.05 to 4.48	18.61 to 22.91
