# Supplementary material for: Isotopic Niche Analysis of Long-Finned Pilot Whales (Globicephala melas edwardii) in Aotearoa New Zealand Waters
Source: Biology (Basel). 2022 Sep 28;11(10):1414. doi: 10.3390/biology11101414 (PMC9598128; doi:10.3390/biology11101414)
Supplement: Supplementary file 1 [file biology-11-01414-s001.zip › Table S4. TA, SEA and SEAc values.pdf]

Table S4. Isotopic niche total area (TA), standard ellipse area (SEA) and standard ellipse area corrected (SEAc) of carbon and nitrogen ($\delta^{13}C$ and $\delta^{15}N$) values for different reproductive status of long-finned pilot whales (*Globicephala melas edwardii*). Data are presented by location of stranding of *G. m. edwardii*.

	Farewell Spit					Stewart Island			
		Male	Female				Male	Female	
	Immature	Mature	Pregnant	Lactating	Resting	Immature	Mature	Pregnant	Resting
TA	2.98	2.23	3.14	0.74	1.49	1.98	0.30	0.38	0.33
SEA	0.79	1.21	1.75	0.64	1.34	0.61	0.23	0.23	0.38
SEAc	0.82	1.33	1.96	0.77	1.67	0.65	0.27	0.27	0.57
